# Supplementary material for: CALINCA—A Novel Pipeline for the Identification of lncRNAs in Podocyte Disease
Source: Cells. 2021 Mar 20;10(3):692. doi: 10.3390/cells10030692 (PMC8003990; doi:10.3390/cells10030692)

Talyan, Filipow et al. 2021

Supplementary Materials and Methods

S1. qPCR

For the detection of lncRNAs' expression in the glomeruli and the whole mouse kidneys, we performed quantitative RT-PCR analyses. Glomeruli isolation was carried out with magnetic beads as described previously [40]. Total RNA was extracted from whole kidney samples and isolated glomeruli using the Direct-zol RNA Kit (Zymo Research). The quantity of extracted RNA was measured using a Qubit 4 Fluorometer (Invitrogen). The cDNA was synthesized from 2 µg of total RNA using the High-Capacity cDNA Reverse Transcription Kit (Applied Biosystem). The qPCR was performed for following targets: *Wt1os*, *4921504A21Rik*, *Gm10824*, *Gm28876* and *Gm26759*, using the custom TaqMan PrimeTime assays (Integrative DNA Technologies) and TaqMan™ Gene Expression qPCR Assay MasterMix (Applied Biosystems), according to the manufacturer's recommendations. The analyses were run with 7900HT Fast Real-Time PCR System (Applied Biosystem), using Sequence Detection Systems Software version 2.4 software (Applied Biosystem). The TaqMan PrimeTime primer and probe sequences are listed in the Table S1. All qPCR reactions were performed under the following conditions: 1 cycle at 95 °C for 10 min; 40 cycles of 95 °C for 15 s, 60 °C for 60 s, and 1 cycle at 72 °C for 10 min. The TaqMan PrimeTime assays spanned an intron to avoid unwanted amplification of genomic DNA. Analysis was performed using the $2^{-\Delta\Delta Ct}$ method, with mouse *POLR2A* as a reference gene. To control for possible contamination, water blanks were placed in the PCR run. An unpaired t-test was used to test statistical significance with P value < 0.05 considered to indicate a statistically significant difference (*P < 0.05; **P < 0.01; ***P < 0.001). The graphs were drawn using GraphPad 6 software.

Primers and probes sequences

Gene	Forward primer	Reverse primer	Probe
Wt1os	TGGCCTTCCCGAGTGTA	CAGGAGGATCGGAAGTTCAAG	CATTCCAGTCCTAGACGGCCTTGC
4921504A21Rik	GTTAGAGAGGTTGAGTTAATGTTTGAG	GTGTTAGGTCCTTGTAGCTGTC	TCCTTACATGCTCCAGAGCACAG
Gm10824	GGCTGGAGTCCTGAGTAGAA	TTTCTGTCATCCCTGCTGTG	ACTGCATCCTCCCAAGATTTATTGCA
Gm28876	CCTTCTATTCCAACACCTCTTCC	GTTGTGCTCCGTGTGAGTTA	ATGGAAGCAAGAGCCCAGTGAAGT
Gm26759	CCAGGAGGAAGAACTACAGAAAT	AGGTGTGTCTGAAATACTAAGAGAA	TGCAGTGTGGAGTACTTGTGTGACC

Detailed information of the RNAscope probes

Gene	Probe name	Cat no.	Targeted region
Wt1os	Mm-Wt1os	577401	264-1227 of ENSMUST00000172701.7
4921504A21Rik	Mm-4921504A21Rik	851511	52-1522 of ENSMUSG00000097626.5
Gm10824	Mm-Gm10824	851521	2-915 of ENSMUST00000220000.1

XLOC_024349	Mm-Novel24	864451	306-1622 of exons: chr8:128503166-128503665:-; chr8:128499486-128500632:-

S2. RNAseq

For the glomerular RNAseq datasets, libraries were prepared with the Illumina TruSeq stranded ribo zero gold protocol. The first step involves the removal of ribosomal RNA using biotinylated target-specific oligos combined with Ribo-Zero rRNA removal beads from 1ug totalRNA input. The Ribo-Zero Human/Mouse/Rat gold kit depletes samples of cytoplasmic and mitochondrial rRNA. Following purification, the RNA is fragmented into small pieces using divalent cations under elevated temperature. The cleaved RNA fragments are copied into first strand cDNA using reverse transcriptase and random primers, followed by second strand cDNA synthesis using DNA Polymerase I and RNase H. These cDNA fragments then have the addition of a single'A' base and subsequent ligation of the adapter. The products are purified and enriched with PCR (20ul template, 15cycles) to create the final cDNA library. After library validation and quantification (Agilent 4200 tape station), equimolar amounts of library were pooled. Pools were quantified by using the Peqlab KAPA Library Quantification Kit and the Applied Biosystems 7900HT Sequence Detection System and sequenced on an Illumina NovaSeq6000 sequencer with PE100 read length.

S3. RNAScope

The RNAscope probes’ catalog numbers and targeted regions are listed in the Table S2. All the procedures were performed according to manufacturer’s guidelines for the FFPE tissue. In brief, FFPE sections were deparaffinized, followed by 10 minutes of hydrogen peroxide pretreatment, incubation in Target Retrieval Reagent for 30 minutes, and permeabilization with protease for 30 minutes at 40 °C. Tissue sections were then incubated with ACD mouse target probes for 2 h at 40 °C. The slides were washed thoroughly using the wash buffer (ACD, Inc.) after each step at room temperature. Amplification steps were performed and diaminobenzidine (DAB) was used as the chromogen. The samples were counterstained using Hematoxylin Solution, Gill No. 1 (Sigma-Aldrich). Positive (mouse *Ppib*) and negative (*DapB*) control probes were included in each experiment. The positive staining was identified based on brown punctate spotting in the nucleus and/or cytoplasm. Representative images were captured using the Slide Scanner Leica SCN400 system (Leica Biosystems) and prepared in Aperio ImageScope 12.4.3 software (Leica Biosystems).

Supplementary figure legends

Supplementary figure 1. Graphical representation of urine albumin-to-creatinine ratios (UCAR) in BALB/c mice 5 days after intravenous injection of adriamycin in comparison to untreated control mice.

Supplementary figure 2. Graphical representation of qPCR data shown in figure 4A. The expression of the shown transcripts was compared between RNA extracted from mouse glomeruli and whole kidneys. The results are representative of three biological replicates, using three technical replicates for whole kidney and two for glomerular RNA. The fold change (glomeruli over whole kidney) was calculated using the delta-delta Ct method with Polr2a as a control gene. An unpaired t-test was used to detect significant differences between expression in glomeruli and whole kidney. ***$p<0.001$, **$p<0.01$, *$p<0.05$

Supplementary figure 3. Localization of *Wt1os* (A), *4921504A21Rik* (B), *Gm10824* (C) and XLOC_024349 (D) by RNAscope. Representative images of glomeruli and tubules analyzed with custom designed RNAscope probes for lncRNAs *Wt1os*, *4921504A21Rik, Gm10824* and XLOC_024349 shown in figure 4A/B. Target lncRNAs were detected with the RNAscope 2.5 HD – brown assay on FFPE mouse kidney tissue sections. Probe binding is visualized as punctate brown dots. Counterstain: hematoxylin (blue). Scale bar: 60 µm.

Supplementary figure 4. Localization of *WT1-AS*, the human homolog of *Wt1os,* shown in figure 4C. A) *WT1-AS* and controls were detected with the RNAscope 2.5 HD – brown assay on FFPE human kidney tissue sections. Probe binding is visualized as punctate brown dots. Counterstain: hematoxylin (blue). Scale bar: 200µm. B) Additional image of human glomeruli and tubules analyzed with the custom designed RNAscope probe for *WT1-AS*.

Supplementary Table legends

Supplementary Table 1. Excel spreadsheet containing the output of all analyses of the RNAseq data performed. Descriptions of the individual columns are provided below. calinca.dieterichlab.org provides an interactive version of this table allowing for both user-defined content and visualization of the data.

Column descriptions

XLOCid - unique gene ID generated by Cufflinks

GeneSymbol - unique abbreviation for Ensembl annotated mouse lncRNA gene or XLOC ID for the novel genes

GenomicCoordinates - Ensembl based genomic locus coordinates

ExonNumber - Number of exons per transcript

TranscriptLength - Length of the transcripts in bases

Podocytes.FPKM - FPKM of lncRNA genes from FACS sorted podocytes (wildtype)

Glomerulus.FPKM - FPKM of lncRNA genes from isolated glomeruli (wildtype)

WholeKidney.FPKM - FPKM of lncRNA genes from whole mouse kidneys (wildtype)

TauMeasure - Tissue specificity index calculated as described by Kryuchkova-Mostacci et al., 2017 (DOI: 10.1093/bib/bbw008). LncRNAs with an index of > 0.8 are considered tissue-specific.

TauClassification - Tissue specificity classification based on TauMeasure (cutoff 0.8), indicates in which tissue the lncRNA is enriched.

Podocyte.Enriched.Transcript – Transcript enriched in podocytes over glomeruli and whole kidney based on Tau analysis

Podocyte.Expressed.Transcript –Transcript showing FPKM > 0 in podocytes

SequenceConservation - sequence homology-based gene conservation analysis, indicates whether the lncRNA is conserved between mouse and human based on sequence homology (BLASTN)

SyntenyConservation - synteny-based gene conservation analysis, indicates whether the gene order of the lncRNA gene surrounding protein coding genes is conserved between mouse and human (CYNTENATOR, Rödelspeger & Dieterich, 2010)

Human.Expressed.GTEX – lncRNA, the homolog of which is found expressed in human kidney cortex (based on an analysis of datasets from the GTEx database)

Wt1.2factor.log2FC - Log_2 fold change of gene expression for 4 and 12 week samples of the $Wt1^{+/-}$ disease model analyzed in a combined fashion using a two-factor model

Wt1.2factor.FDR - Combined False Discovery Rate value for 4 and 12 week samples of the $Wt1^{+/-}$ disease model analyzed in a combined fashion using a two-factor model

Wt1.4.FDR - False Discovery Rate values for the 4 week samples of the $Wt1^{+/-}$ disease model

Wt1.4.log2FC - Log_2 fold change of gene expression for the 4 week samples of the $Wt1^{+/-}$ disease model

Wt1.12.FDR - False Discovery Rate values for the 12 week samples of the $Wt1^{+/-}$ disease model

Wt1.12.log2FC - Log_2 fold change of gene expression for the 12 week samples of the $Wt1^{+/-}$ disease model

Pod.R231Q_A286V.12.FDR - False Discovery Rate values for the 12 week samples of the $Podocin^{R231Q\_A286V}$ disease model

Pod.R231Q_A286V.12.log2FC – Log_2 fold change of gene expression for the 12 week samples of the $Podocin^{R231Q\_A286V}$ disease model

Pod.R231Q_A286V.2Factor.FDR – Combined False Discovery Rate value for 4 and 12 week samples of the Podocin^{R231Q_A286V} disease model analyzed in a combined fashion using a two-factor model

Pod.R231Q_A286V.2Factor.log2FC – Log_2 fold change of gene expression for 4 and 12 week samples of the Podocin^{R231Q_A286V} disease model analyzed in a combined fashion using a two-factor model

Pod.R231Q_A286V.4.FDR – False Discovery Rate values for the 4 week samples of the Podocin^{R231Q_A286V} disease model

Pod.R231Q_A286V.4.log2FC – Log_2 fold change of gene expression for the 4 week samples of the Podocin^{R231Q_A286V} disease model

Adriamycin.FDR - False Discovery Rate values for the adriamycin disease model

Adriamycin.log2FC - Log_2 fold change of gene expression for the adriamycin disease model

DE.Adriamycin – Classification (yes/no) of differentially expressed genes in the adriamycin dataset (FDR < 0.05 in at least one of the three analyses)

DE.Podocin - Classification (yes/no) of differentially expressed genes in the Podocin^{R231Q_A286V} dataset (FDR < 0.05 in at least one of the three analyses)

DE.WT1 – Classification (yes/no) of differentially expressed genes in the *Wt1*$^{+/-}$ dataset (FDR < 0.05 in at least one of the three analyses)

Podocyte.Enriched.Boerries.FDR - False Discovery Rate values for lncRNA enrichment in podocytes, calculated using data from sorted glomerular cells (Boerries et al., Kidney Int. 2013)

Podocyte.Enrichment.Boerries.log2FC - Log_2 fold change of lncRNA enrichment in podocytes, calculated using data from sorted glomerular cells (Boerries et al., Kidney Int. 2013)

Podocyte.Enriched.Boerries – Classification (yes/no) of lncRNAs enriched in podocytes based on the datasets from Boerries et al., Kidney Int. 2013

Novel – Indicates whether the lncRNA is novel or known (annotated)

Chung.control1.pValue – p value of wilcox.test (GSM4409507) for podocyte enrichment in scRNA sequencing by Chung et al., 2020

Chung.control2.pValue – p value of wilcox.test (GSM4409508) for podocyte enrichment in scRNA sequencing by Chung et al., 2020

Chung.control3.pValue – p value of wilcox.test (GSM4409509) for podocyte enrichment in scRNA sequencing by Chung et al., 2020

scRNAseq.Chung.confirmed – lncRNA expression confirmed (yes/no) by scRNA sequencing (Chung et al., 2020)

NcRNA.GeneID.HSA – Ensembl gene ID of conserved, annotated human lncRNA gene

GeneID.EnsemblHSA.ProteinCoding – Gene identifier of known human protein coding anchor gene annotated in Ensembl database in our synteny assessment

GeneID.EnsemblMMU.ProteinCoding – Gene identifier of known mouse protein coding anchor gene annotated in Ensembl database in our synteny assessment

GeneSymbol.HSA - unique abbreviation for Ensembl annotated human lncRNA gene

Distance.MMU – Distance in base pairs between lncRNA and the closest protein-coding gene

Distance.HSA – Distance in base pairs between human homolog lncRNA and the closest protein-coding gene

ClosestNcTx – A human noncoding gene closest to the human protein homolog in our synteny assessment.

NcRNASymbol – Gene symbol of conserved, annotated human lncRNA gene

ConservedHumanTX - Ensembl transcript identifier of human conserved lncRNA as identified by SequenceConservation

Transcript.ID - unique transcript ID generated by Cufflinks

Type – Transcript biotype classification according to Ensembl

Supplementary figure 1:

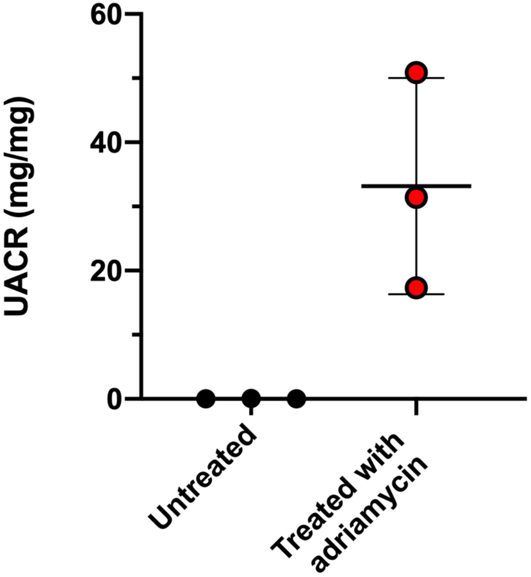

Supplementary figure 2:

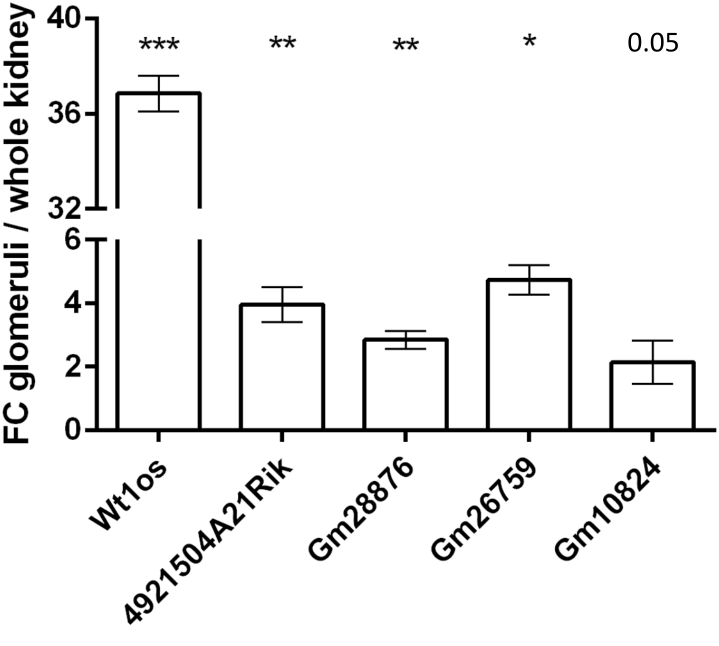

Supplementary figure 3

A) *Wt1os*

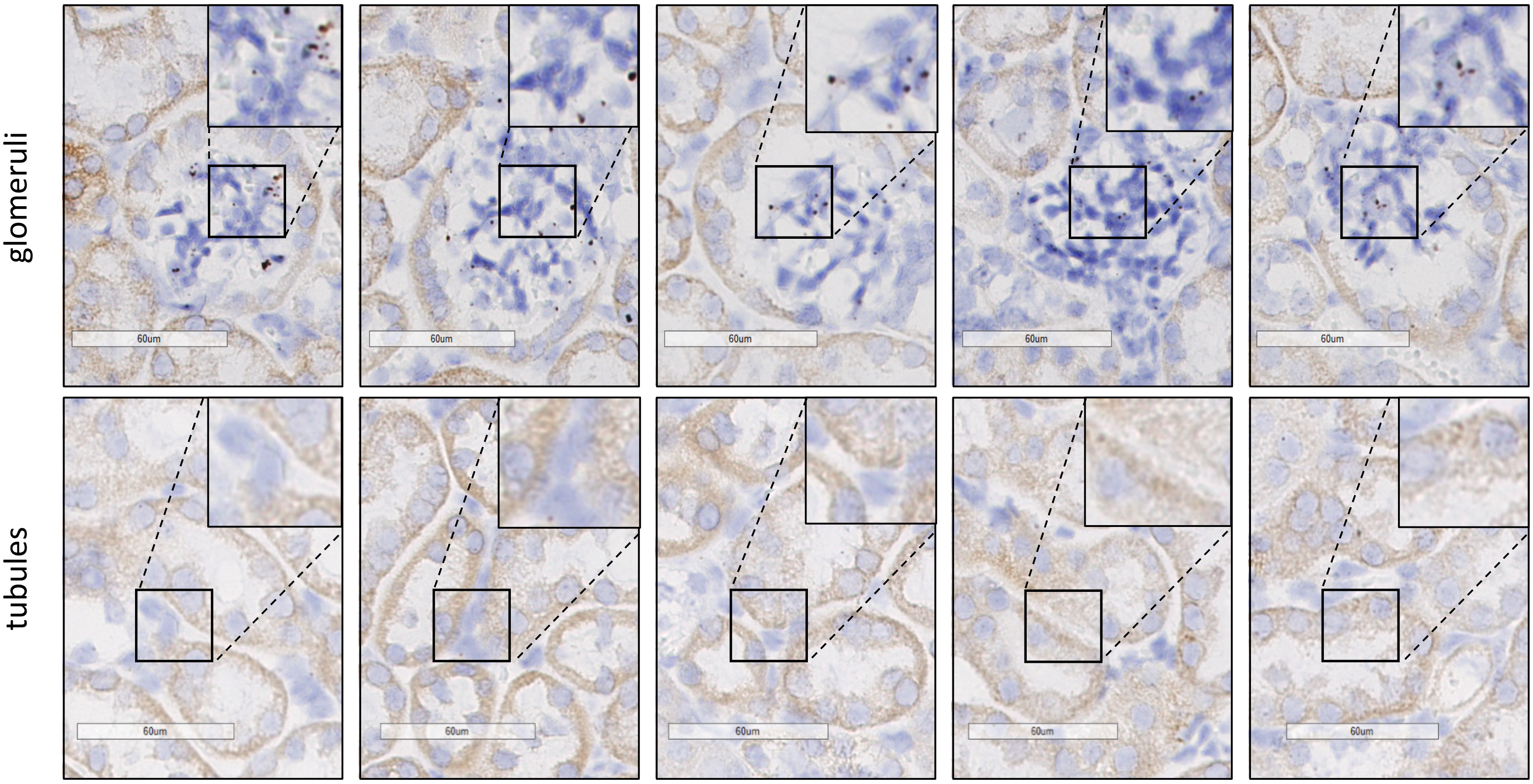

B) *4921504A21Rik*

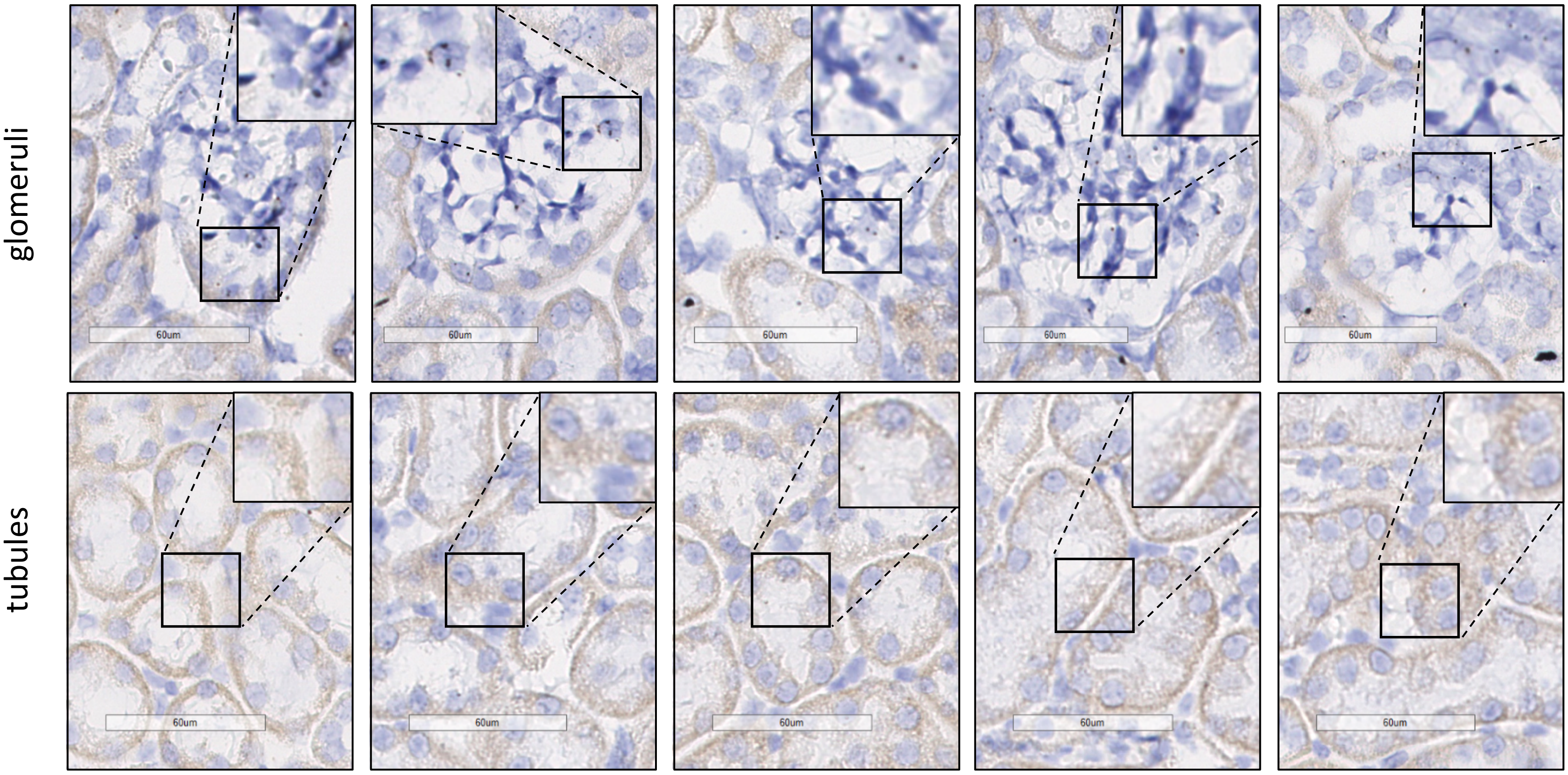

C) Gm10824

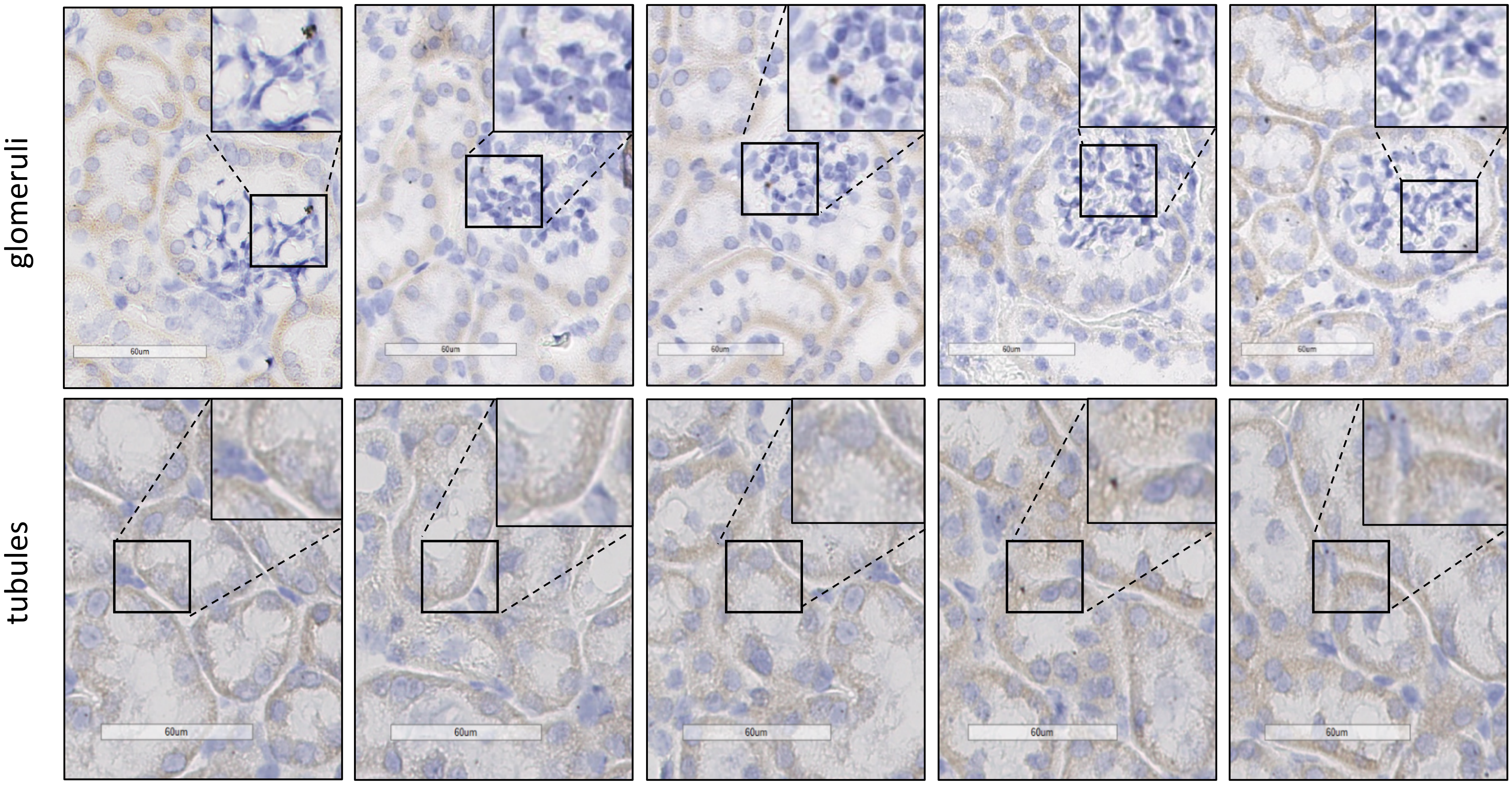

D) XLOC_024349

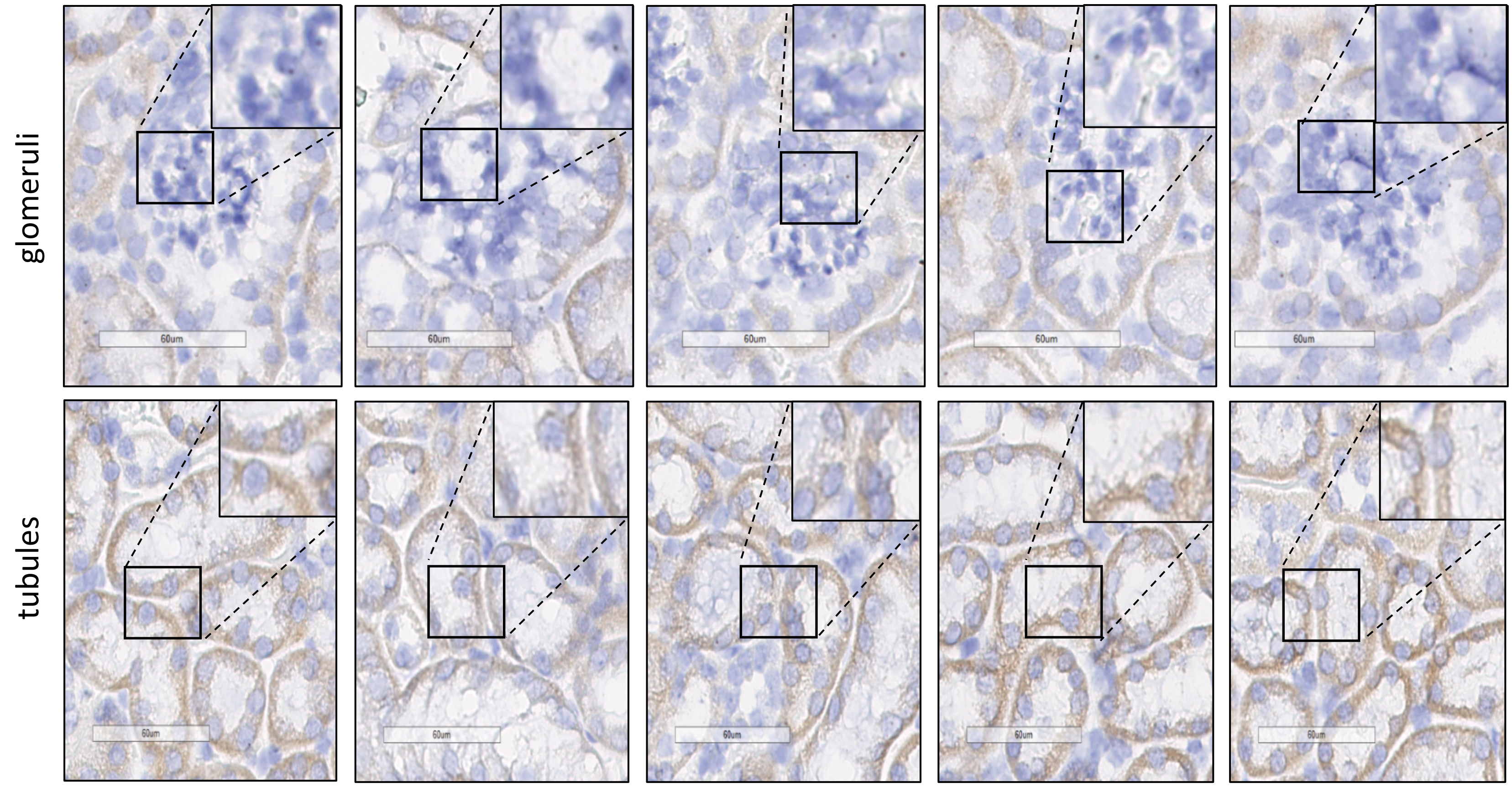

Supplementary figure 4

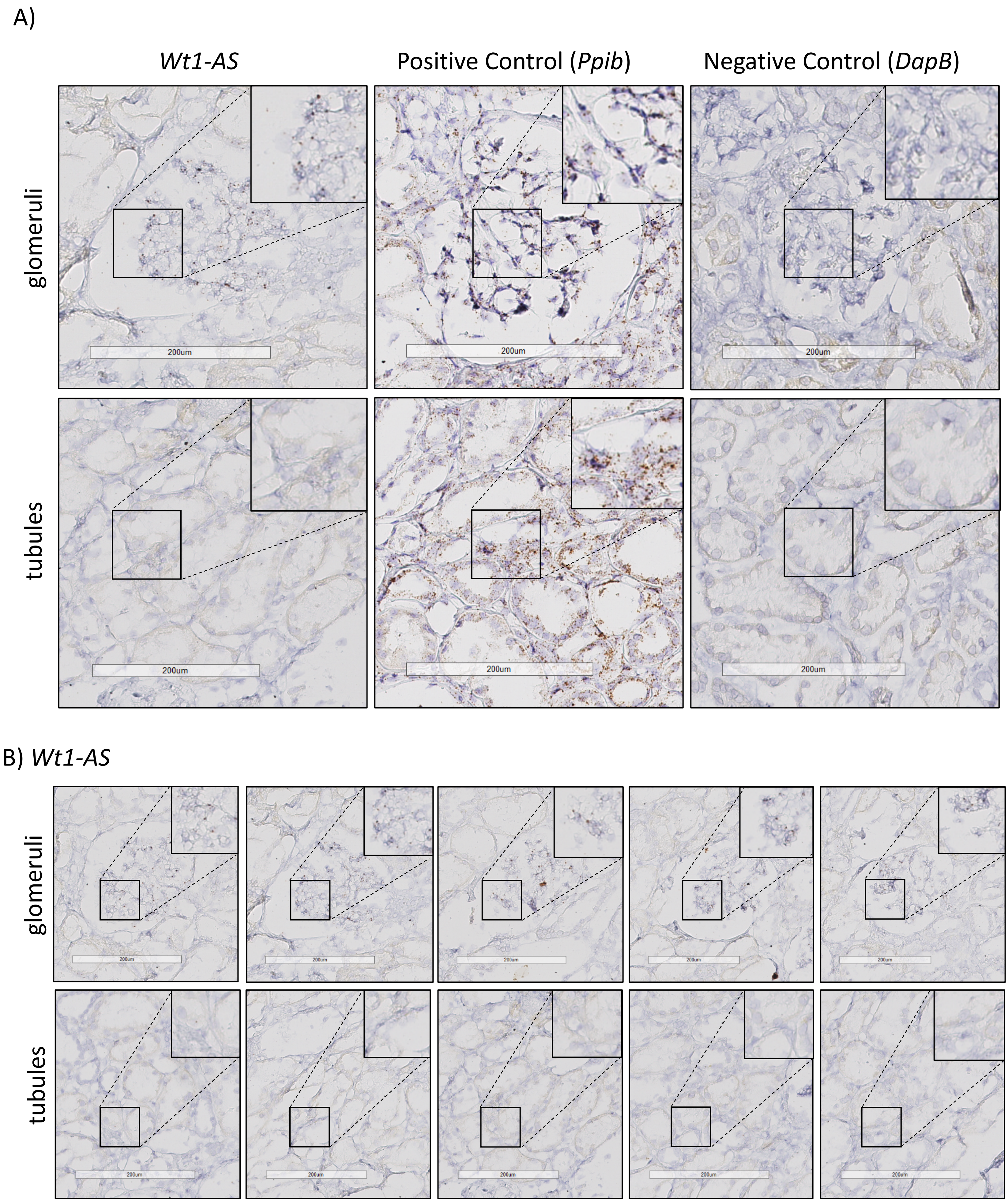

Supplement: Supplementary file 1 [file cells-10-00692-s001.pdf]
